# Study of ZHENG differentiation in Hepatitis B-caused cirrhosis: a transcriptional profiling analysis

**DOI:** 10.1186/1472-6882-14-371

**Published:** 2014-10-03

**Authors:** Yi-Yu Lu, Qi-Long Chen, Yan Guan, Zhi-Zhong Guo, Hui Zhang, Wei Zhang, Yi-Yang Hu, Shi-Bing Su

**Affiliations:** Research Center for Traditional Chinese Medicine Complexity System, Shanghai University of Traditional Chinese Medicine, 1200 Cailun Road, Pudong, Shanghai, 201203 China; Longhua Hospital, Shanghai University of TCM, Shanghai, 200126 China; Shuguang Hospital, Shanghai University of TCM, Shanghai, 201203 China

**Keywords:** ZHENG differentiation, Transcriptional profiling, Differentially expressed genes, Gene co-expression, Hepatitis B**-**caused cirrhosis

## Abstract

**Background:**

In traditional Chinese medicine (TCM) clinical practice, ZHENG (also known as TCM syndrome) helps to understand the human homeostasis and guide individualized treatment. However, the scientific basis of ZHENG remains unclear due to limitations of current reductionist approaches.

**Methods:**

We collected the leukocyte samples of three hepatitis B-caused cirrhosis (HBC) patients with dampness-heat accumulation syndrome (DHAS) and three HBC patients with liver depression and spleen deficiency syndrome (LDSDS) for microarray analysis. We generated Gene-Regulatory-Networks (GeneRelNet) from the differentially expressed genes (DEGs) of microarray date. Core genes were validated using anther independent cohort of 40 HBC patients (20 DHAS, 20 LDSDS) with RT-PCR.

**Results:**

There were 2457 mapped genes were differentially expressed between DHAS and LDSDS (Fold change **≥** 2.0, *P* < 0.05). There were markedly different genes co-expression patterns in DHAS and LDSDS. Furthermore, three differential co-expression genes including purine nucleoside phosphorylase (*PNP*); aquaporin 7 (*AQP7*) and proteasome 26S subunit, non-ATPase 2 (*PSMD2*) were screened by GeneRelNets, and their mRNA expressions were further validated by real time RT-PCR. The results were consistent with microarray. The *PNP* (P = 0.007), *AQP7* (P = 0.038) and *PSMD2* (P = 0.009) mRNA expression is significant difference between DHAS and LDSDS using the non-parametric test. Furthermore, we constructed an mRNA panel of *PNP*, *AQP7* and *PSMD2* (PAP panel) by logistic regression model, and evaluated the PAP panel to distinguish DHAS from LDSDS by area under the receiver operating characteristic curve (AUC) analysis, which showed a higher accuracy (AUC = 0.835). Gene ontology (GO) analysis indicated that the DHAS is most likely related to system process while the functions overrepresented by LDSDS most related to the response to stimulus.

**Conclusions:**

This study suggested that there are particular transcriptional profiles, genes co-expressions patterns and functional properties of DHAS and LDSDS, and *PNP*, *AQP7*, and *PSMD2* may be involved in ZHENG differentiation of DHAS and LDSDS in HBC.

**Electronic supplementary material:**

The online version of this article (doi:10.1186/1472-6882-14-371) contains supplementary material, which is available to authorized users.

## Background

Chronic hepatitis B virus (HBV) infection is a serious health problem because its worldwide distribution and it has potential adverse sequelae such as cirrhosis and hepatocellular carcinoma (HCC) [1, 2]. It was estimated that more than 200,000 chronic HBV carriers die of liver cirrhosis each year globally [3]. Since HBV replication may persist after the development of chronic hepatitis B (CHB) [2], hepatitis B**-**caused cirrhosis (HBC) is easily happen. Moreover, HBC increases the risk of developing HCC by at least 40 times over the risk of an average person [4].

Traditional Chinese medicine (TCM) is extensively used for treatment of liver disease in China, with the great advantages in early intervention, combination therapies, personalized medicine, *etc*. TCM treatment is based on ZHENG (also known as TCM syndrome) differentiation, namely, discerning patterns of imbalances within the body and between the body and the environment by the analysis of symptoms and signs of patients. It is the key principle of TCM. Since ZHENG differentiation depended on clinical observation and TCM practitioner’s experience, it would be subjective and unrepeatable. The phenotype-oriented diagnosis and therapy in TCM needs to be objective and quantified evaluation for the inheritance [5].

TCM has long been practiced as an empirical and holistic medicine, which happens to share the same concept with the systems biological medicine. The advent of high-throughput technologies, such as transcript microarray chips, has allowed simultaneous interrogation of multiple molecular components at any given time. Advances in gene expression profiling have been used to identify key differentially expressed genes (DEGs) and pathways in HBV-related patients with different ZHENGs. In the previous study, we reported that the molecular mechanism of “same TCM syndrome for different diseases and different TCM syndrome for same disease” [6]. Besides, we integrated the CHB gene expression with topological features of different ZHENGs resulted in enhanced diagnosis of CHB. The miRNA-target network analysis elucidated dysfunctional interactions among genes and miRNAs-target genes of three different ZHENGs [7]. Above all, the transcriptional profiles of ZHENG differentiation in HBC could be interpreted using the systems-omics approach.

The dampness-heat accumulation syndrome (DHAS) and the liver depression and spleen deficiency syndrome (LDSDS) are the major ZHENGs in HBC [8]. In this study, we demonstrated that the transcriptional profiles and the functional properties are significantly different in DHAS and LDSDS. Besides, the differential expression or co-expression genes could be the potential biomarkers for ZHENG differentiation with DHAS and LDSDS in HBC patients.

## Methods

### Samples and RNA extraction

Morning fasting venous blood samples of total 46 HBC patients (23 DHAS, 23 LDSDS) were obtained from Shuguang Hospital and Longhua Hospital in Shanghai, China. Six HBC patients (3 DHAS, 3 LDSDS) were used for microarray analysis. Another independent cohort of 40 HBC patients (20 DHAS, 20 LDSDS) was used as validation group. The research protocol was approved by the respective institutional review boards, and informed consent was obtained for all study participants. The study was approved by the Official Ethics Committee of the Shanghai University of TCM and written informed consent to participate in the study was obtained from all subjects included. Diagnosis standard of cirrhosis is referred to “Chronic hepatitis B prevention and treatment guidelines” [9]. The TCM ZHENG types were identified by three chief or deputy physicians, according to “evaluation criteria of the clinical diagnosis, drug efficacy, and ZHENG differentiation for cirrhosis (pilot program)” [8]. All the outpatients were diagnosed by attending TCM physicians at the first time and then identified by three chief TCM physicians, who were consistently diagnosed as DHAS or LDSDS by all of the physicians were enrolled in this study [10, 11]. The patients with other hepatotropic virus hepatitis, chronic severe hepatitis, serious primary disease or pregnancy were excluded. The exclusion criterion of HBC: (1) cases complicated with other hepatotropic virus; (2) age < 18 or age > 65; (3) associated with serious primary disease of heart, kidney, lung, endocrine, blood, metabolic and gastrointestinal; (4) psychotic patients; (5) pregnant or lactating women. Samples of three DHAS, three LDSDS and three normal control were used to microarray detection. Another independent twenty DHAS and twenty LDSDS in HBC patients were used to verify the accuracy of the results. The characteristics of the study participants are presented in Table 1. The leukocyte from blood samples were isolated with Ficoll optimized density gradient separation [12] and saved at -80°C.

**Table 1:** **Clinical parameters of subjects**

Characteristics	DHAS (mean ± SD)	LDSDS (mean ± SD)
**n = 46**	23	23
**Age (years)**	53.2 ± 8.1	52.9 ± 6.7
**Gender (male)**	13	15
**ALT (U/L)**	33.8 ± 14.8	33.2 ± 18.0
**AST (U/L)**	42.6 ± 29.7	54.1 ± 39.6
**AFP (ng/mL)**	7.3 ± 5.9	9.6 ± 10.0
**HA (UG/L)**	124.3 ± 121.3	152.1 ± 126.8
**TBIL (μmol/L)**	20.1 ± 11.4	25.4 ± 16.3
**DBIL (μmol/L)**	4.1 ± 4.4	6.6 ± 6.1
**GGT (IU/L)**	48.9 ± 28.9	52.0 ± 30.5
**ALb (g/L)**	41.0 ± 8.0	41.0 ± 6.1

Total RNA was extracted using a “two-step” protocol as described previously [6]. Total RNA of leukocyte from the whole blood was extracted using TRIzol® Reagent (Invitrogen, Carlsbad, CA, US) and saved at -80°C. A Quality Control was carried out with NanoDrop ND-1000.

### Microarrays and data analysis

The biotinylated cDNAs were hybridized to NimbleGen Homo sapiens 12 × 135K Array (Roche, CAT No. A6484-00-01). This product analyzes the expression level for over 135,000 transcripts and variants, including 44049 well-substantiated human genes.

Microarray data preprocessing was performed using the Gene-Pix software. Raw expression data were log2 transformed and normalized by quantile normalization. Probes were considered robustly expressed if Signal/Noise (SNR) > 2.

The t-test function in R software was used to select difference expressed gene (threshold: P value < 0.05) in ZHENGs of DHAS and LDSDS. They were both compared with normal control. Heatmap analysis which executed in Cluster 3.0 was computing the hierarchical clustering in both rows and columns according to the set of gene values and drawing a color image as a visible result. Gene Ontology (GO) was analyzed using a DAVID online analysis tool (http://david.abcc.ncifcrf.gov).

We used a stepwise logistic regression model to combine diagnostic mRNA markers based on the data obtained by qRT-PCR. The predicted probability of distinguishing of DHAS and LDSDS was used as a surrogate marker to construct a receiver operating characteristic (ROC) curve. The AUC was used as an accuracy index for evaluating the diagnostic performance of the selected mRNA panel. All tests were two-tailed and *P* < 0.05 was considered statistically significant.

### Gene-Regulatory-Networks (GeneRelNet) construction

GeneRelNets [13] were presented to find the interactions among genes, indicating the gene co-expression relationship. GeneRelNets was constructed by DEGs that obtained by comparing the DHAS or LDSDS. They were built according to the normalized signal intensity of specific expression genes. For each pair of genes, we calculate the Pearson correlation and choose the significant correlation pairs to construct the network.

In network analysis, Degree centrality, Clustering coefficient and Core genes were analyzed. Degree centrality, defined as the link numbers one node has to the other, is the most simplest and important measures of the centrality of a gene within a network that determine the relative importance [14]. Clustering coefficient is a measure of degree to which nodes in a graph tend to cluster together. The higher clustering coefficient of an individual gene, the higher is the affection among its neighbor genes [15]. The purpose of network structure analysis of each ZHENG is to locate differential co-expression genes, which connect most adjacent genes and have biggest degrees with high Clustering coefficient. While considering different networks, the Core genes were determined by the degree differences between two class samples [16]. They always own the biggest degree differences and selected from the located differential co-expression genes.

### Real-time RT-PCR

The cDNAs were synthesized by the Invitrogen First-Strand cDNA Synthesis kits (Invitrogen, Carlsbad, CA, US). Samples will be qualified for further processing if the A260/A280 spectrophotometeric ratio is between 1.8 and 2.1. One μg of total RNA was transcribed into cDNA in a 20 μL reaction volume.

Real-time RT-PCR was used to verify the differential expression of three genes that detected by the NimbleGen GeneChip. The primers used are listed in Table 2. Each real-time RT-PCR reaction (in 25 μL) contained 2 × SYBR Green Real-time RT-PCR Master Mix, 0.4 μM primers, and 0.5 μL of template cDNA. The cycling conditions consisted of an initial, single cycle of 5 min at 95°C, followed by 40 cycles of 30 sec at 95°C, 30 sec at 54°C, 15 sec at 72°C, and fluorescence acquisition at 83°C for 1 sec. The cDNA was synthesized using reverse transcriptase (TOYOBO, Osaka, Japan), oligo (dT) and random primers with 5 μg RNA from the same samples as those used in the microarray. The PCR amplifications were performed in duplicate for each sample. The gene expression levels were quantified relative to the expression of β-actin by employing an optimized comparative Ct (ΔΔCt) value method. The differences in gene expression levels between groups were compared using the Student's t-test. A *p* value < 0.05 was considered significant.

**Table 2:** **Validated genes and their primer sequences by real-time RT-PCR**

Gene symbol	Genebank	Forward primer	Reverse primer
*PNP* (purine nucleoside phosphorylase)	NM_000270	GAGCCCGTGCCCTACCACAC	GCAGAACTGAGCCCCTCGGAA
*AQP7* (aquaporin 7)	NM_001170	TGAGAAGCCCCCAAGGCGGA	GCCTGTGCCCGGATGCTTGA
*PSMD2* (proteasome 26S subunit, non-ATPase 2)	NM_002808	TGCGGCCATTGCCAGTGTCT	GTTCCCCGTGGGCCAACAACA
*ACTB* (β-actin)	NM_001101.3	ACAGAGCCTCGCCTTTGCCG	ACATGCCGGAGCCGTTGTCG

## Results

### Identification of core genes of DHAS and LDSDS in HBC

**Figure 1:**
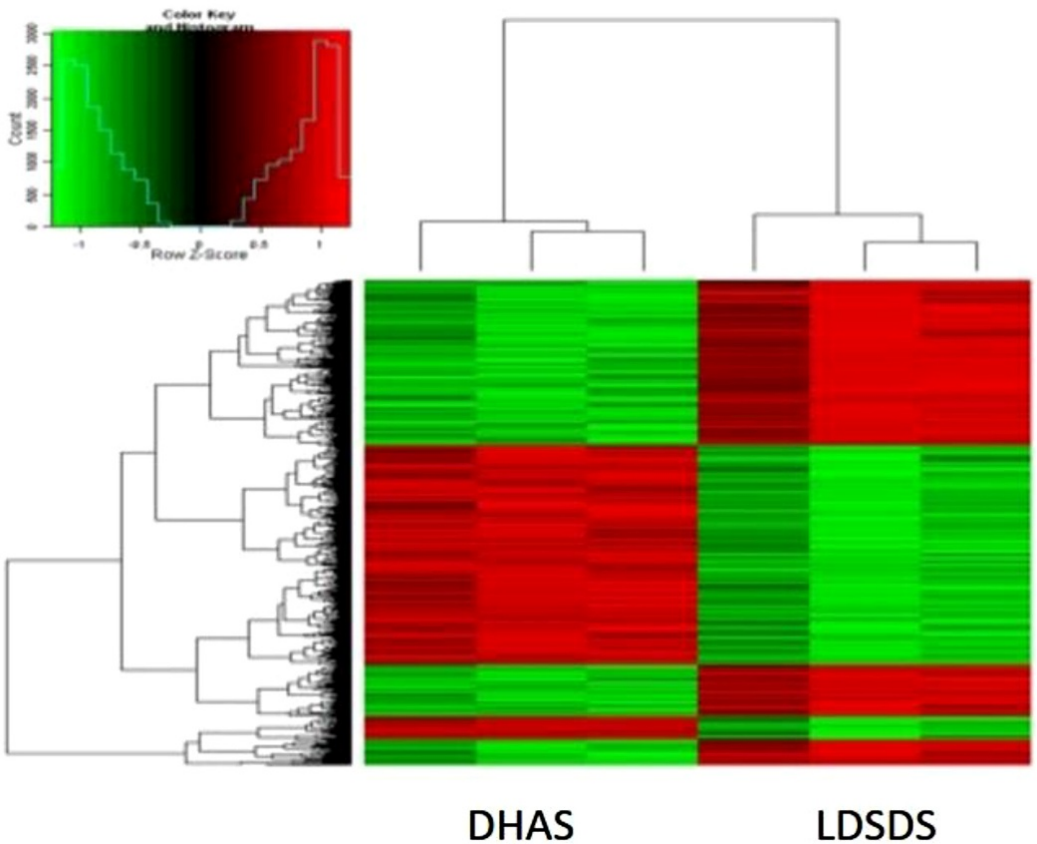
**Hierarchical clustering of DEGs.** DEGs of DHAS vs. LDSDS obtained after comparing DHAS/normal control and LDSDS/normal control. An unsupervised hierarchical clustering of DEGs of DHAS and LDSDS showing significantly differential expression revealed two distinct clusters. Log ratio scale bar for the Treeview color change was also shown.

We analyze the DEGs of DHAS and LDSDS from the microarrays data. Both samples from DHAS and LDSDS patients were compared with normal controls and then compared to each other for the array analysis. DEGs were defined as |foldchange | ≥ 2, *p* < 0.05. 3133 genes were identified after comparison of DHAS and normal control. 3627 genes were identified after comparison of LDSDS and normal control. Then the DHAS group compared with LDSDS group. After the two steps of DEGs selection, a total of 2457 mapped genes were identified to be differentially expressed in DHAS compared to LDSDS with |foldchange (DHAS/LDSDS) | ≥ 2, *p* < 0.05. (Figure 1), among which 1855 genes were up-regulated and 602 genes were down-regulated.

**Figure 2:**
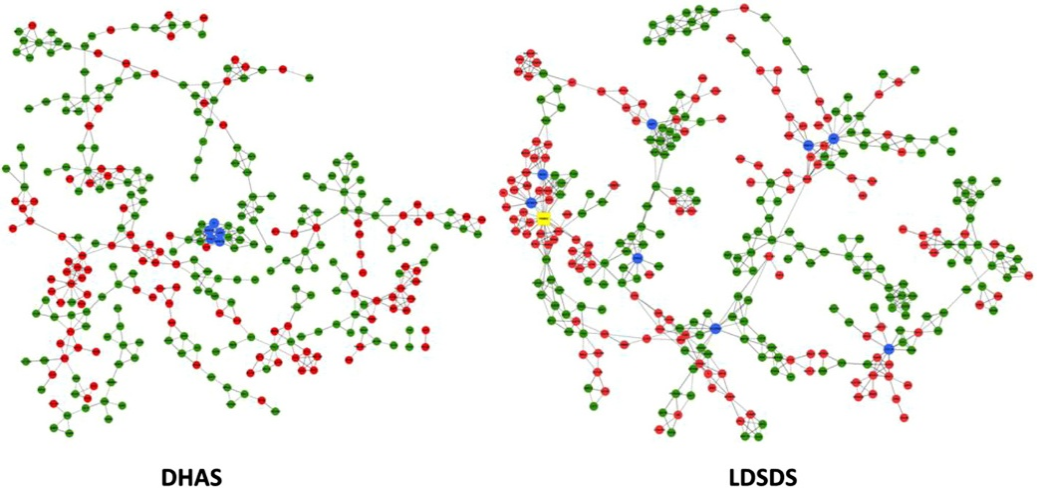
**DHAS and LDSDS GenRelNets.** Left is DHAS GenRelNet and right is LDSDS GenRelNet. The points (nodes) in the pictures stand for genes. Red points represent up-regulated genes; green points represent down-regulated genes. The lines stand for relationships of regulation, solid line means positive correlation; long-dash line means negative correlation. Blue points is screened genes, yellow point is *PSMD2*.

Based on the DEGs of DHAS and LDSDS (Additional file 1), GeneRelNets were constructed, respectively (Figure 2). The structure of GeneRelNet reflects the situation of genes co-expressions, which is interaction among genes. There were markedly different genes co-expression patterns in DHAS and LDSDS (Figure 2). Furthermore, the genes co-expressions analysis was used to screen the candidate genes that may involve in the differentiation of DHAS and LDSDS.

**Figure 3:**
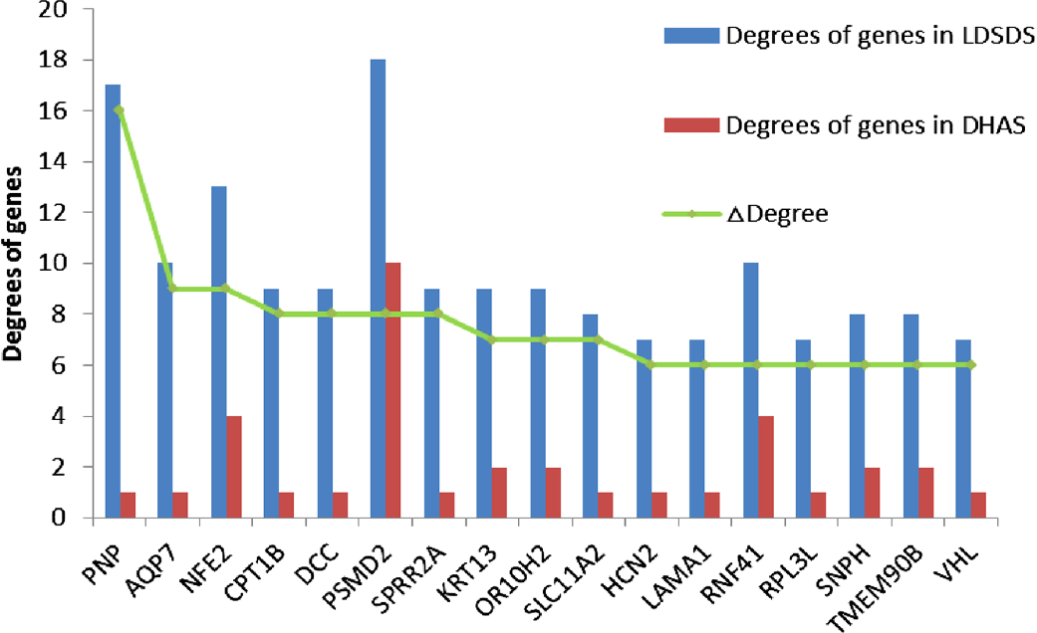
**Differential co-expression genes in DHAS and LDSDS.** Seventeen differential co-expression genes in DHAS and LDSDS with ΔDegree ≥ 5 were listed. Red bar, degrees of genes in DHAS GenRelNet; Blue bar, degrees of genes in LDSDS GenRelNet; Green line, the Δ degree which represents the degree difference of core genes in DHAS and LDSDS.

In network analysis, the differential co-expressed genes with higher Degree Centrality (degree ≥ 10) and higher Clustering Coefficient (Clustering Coefficient ≥ 0. 2) were considered to have important regulation and control ability. They were located from GeneRelNet of DHAS or LDSDS for ZHENG differentiation. Core genes in DHAS and LDSDS had higher degree differences (ΔDegree) (Figure 3). In this study, the differential co-expression genes were defined with ΔDegree ≥ 8. Core genes were *PNP* (ΔDegree, 16), *AQP7* (ΔDegree, 9), *NFE2* (ΔDegree, 9) and *PSMD2* (ΔDegree, 8) (Table 3).

**Table 3:** **Analysis of core genes in DHAS and LDSDS GeneRelNets (ΔDegree ≥ 8)**

Gene	Description	Style	DHAS degree	LDSDS degree	ΔDegree
***PNP***	Purine nucleoside phosphorylase	down	17	1	16
***AQP7***	Aquaporin 7	up	10	1	9
***NFE2***	Nuclear factor (erythroid-derived 2), 45 kDa	down	13	4	9
***PSMD2***	Proteasome (prosome, macropain) 26S subunit,non-ATPase, 2	down	18	10	8

Since *PNP* and *AQP7* were the top differential co-expression genes, and *PSMD2* had the highest Degree in both DHAS and LDSDS. They were chosen to verify the transcript expressions and compare the difference between DHAS and LDSDS in HBC.

### Validation of core genes in DHAS and LDSDS GeneRelNets

**Figure 4:**
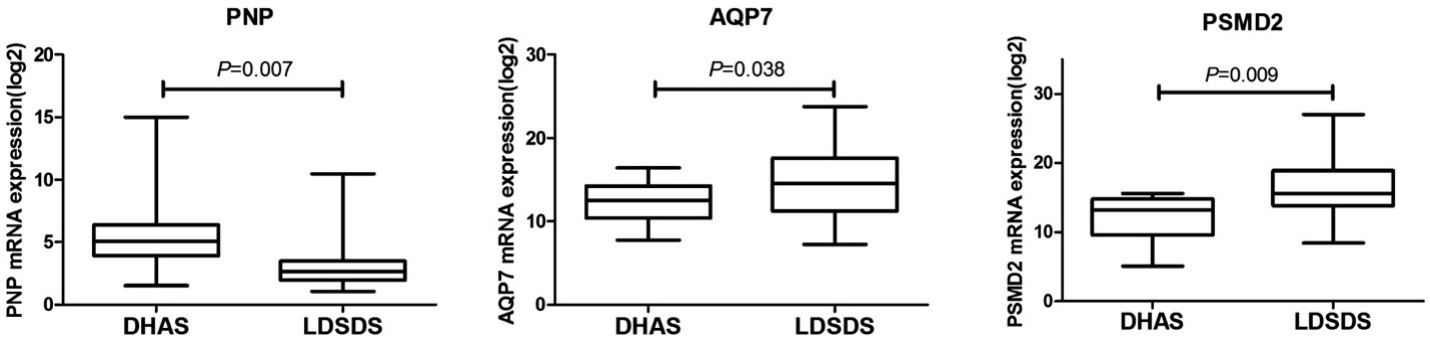
**mRNAs expression of** ***PNP*** **,** ***AQP7*** **and** ***PSMD2*** **.** *PNP*, *AQP7* and *PSMD2* mRNAs expression were tested by real time RT-PCR, and the value of mRNA expressions were log 2-transformed with negative values set to 1. DHAS, n = 20; LDSDS, n = 20. Mann–Whitney U test.

Following the computational analyses of GeneRelNets, quantitative RT-PCR was carried out using a cohort of independent samples of DHAS (n = 20) and LDSDS (n = 20) in HBC. The expression result of *PNP*, *AQP7* and *PSMD2* was consistent with the results from microarrays. At the transcript expression level, statistical significance was noted for *AQP7* and *PSMD2* (Figure 4). *PNP* transcript levels were elevated significantly (P = 0.007) in DHAS group compared with LDSDS group. *AQP7* (P = 0.038) and *PSMD2* (P = 0.009) expression levels were significantly elevated in DHAS group compared to LDSDS group (Figure 4).

### Sensitivity and specificity of core genes of DHAS and LDSDS for ZHENG differentiation

**Figure 5:**
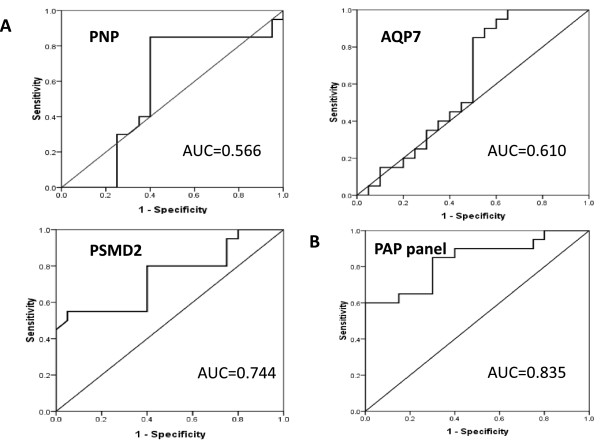
**ROC curves for DHAS and LDSDS differentiation in HBC.** ROC curves were generated using mRNA expression data with DHAS (n = 20) and LDSDS (n = 20) in HBC. **A**, AUC of *PNP*, *AQP7* and *PSMD2* were 0.566, 0.610 and 0.744, respectively. **B**, AUC of mRNA panel of combination of *PNP*, *AQP7* and *PSMD2* (PAP panel) was 0.835.

The levels of *PNP*, *AQP7* and *PSMD2* mRNA expressions between DHAS and LDSDS were compared. As shown in Figure 5, AUC of *PNP*, *AQP7* and *PSMD2* were 0.566 (P = 0.473, 95% CI, 0.372 to 0.761), 0.702 (P = 0.234, 95% CI, 0.426 to 0.794) and 0.767(P = 0.008, 95% CI, 0.587 to 0.900) respectively (Figure 5A).

Furthermore, we applied a stepwise logistic regression model to combine the three core genes to distinguishing DHAS from LDSDS. The predicted probability from the logit model based on the three mRNA panel (PAP panel). The logit model (P = LDSDS) = -0.634 + 13.701 *PNP*-230.17 *AQP7*-512.468 *PSMD2* was used to construct the ROC-curve. The diagnostic performance for the established mRNA panel was evaluated by using ROC analysis. The AUC for the PAP panel was 0.835 (P = 0.000, 95% CI, 0.708 to 0.962, Figure 5B).

### Functional properties of DEGs between DHAS and LDSDS in HBC

**Figure 6:**
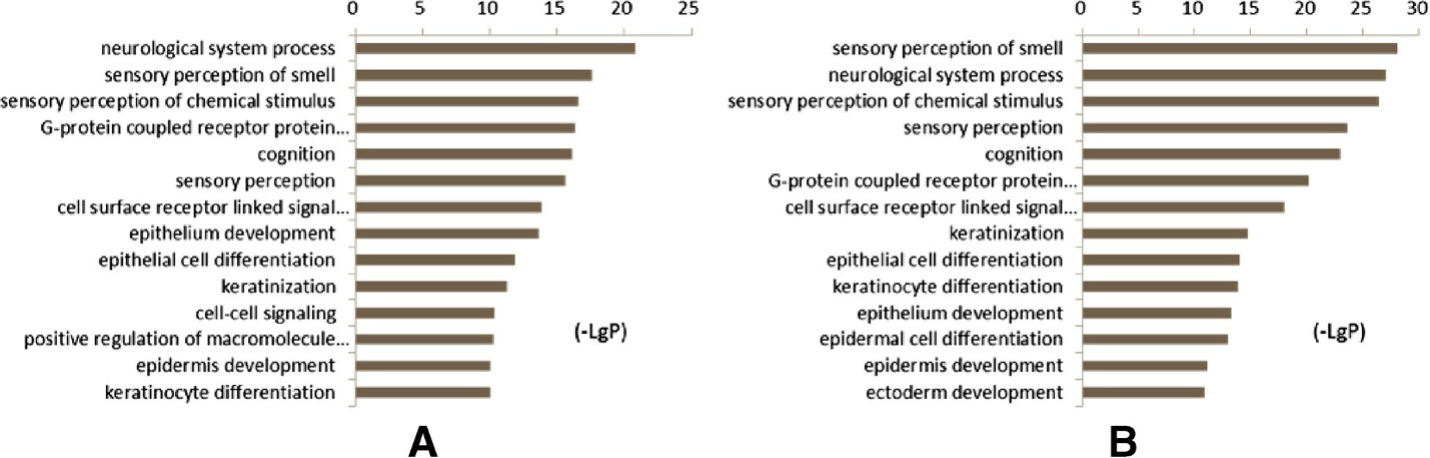
**GO enrichment analyses based on the differentially co-expressed genes of DHAS and LDSDS. A**, GO category based on the biological process for differentially co-expressed genes of DHAS. **B**, GO category based on the biological process for differentially co-expressed genes of LDSDS. - LgP is the base-10 logarithm of the p value.

To further determine differential co-expression genes in DHAS and LDSDS involved in the cellular behavior, we conducted a GO enrichment analysis and searched for functional categories.The results showed that these differential co-expression genes were enriched for neurological system process and sensory perception in DHAS. By contrast, the functions overrepresented by LDSDS samples related to the sensory perception of smell, sensory perception of chemical stimulus, response to stimulus, neurological system process, and cognition, etc. Illustrative gene function diagrams corresponding to DHAS and LDSDS are given (Figure 6), respectively.

## Discussion

TCM ZHENG differs significantly in HBC patients [17]. DHAS and LDSDS are two typical TCM ZHENGs with different clinical phenotype in HBC [8]. In TCM, DHAS is recognized to dampness-heat accumulation. It represents accumulation of dampness-heat in the liver and gallbladder resulting in impaired bile flow and downward pouring of dampness-heat; LDSDS is recognized to the dampness and heat accumulated in liver and gallbladder. It represents a syndrome marked by hypochondriac and abdominal painful distension, depressed mood, frequent sighing, anorexia, uncomfortable loose bowels or alleviation of abdominal pain after defecation, borborygmi with flatus and white slimy tongue coating, the same as the syndrome of liver stagnation and spleen deficiency, also known as the liver-spleen disharmony syndrome [18]. It was the first time to stratify the DHAS and LDSDS of HBC patients with the transcriptional technology.

Elucidating which genetic factors contribute to the ZHENGs differentiation may have important therapeutic implications in TCM practice. High-throughput, genome-wide analytical technologies, especial expression microarrays could be both accurate and precise when properly implemented. It has been used for disease classification and identification of causal mechanisms in different human liver diseases, such as alcoholic liver disease [19], fatty liver disease [20], HCV-associated liver disease [21], liver cancer [22], and even liver tumor metastases [23]. However, it is lack of microarray-based diagnostic algorithm for ZHENG differentiation in HBC.

Since TCM is holistic and systems medicine [24], TCM ZHENG differentiation may be demonstrated through the investigation in gene regulated network. In this study, we identified the whole gene expressions of DHAS and LDSDS in HBC, and we found the significant difference in gene expression patterns between DHAS and LDSDS (Figure 1). Moreover, we propose using co-expression-based gene networks (GeneRelNets) to identify the biomarkers that are associated with DHAS and LDSDS syndromes. In GeneRelNets, the links between genes (nodes) were determined by the extent of their correlated pattern of expression across multiple genes and a variety of biological relationships. Since the DHAS and LDSDS represent the different ZHENGs of HBC, the GeneRelNets analysis showed the markedly different genes co-expressions and their patterns (Figure 2), and there were differential co-expression genes (Figure 3). The degree centrality difference of two GeneRelNets in DHAS and LDSDS indicated a possibility of distinguishing DHAS and LDSDS.

The differential co-expression genes of *PNP*, *AQP7* and *PSMD2* between DHAS and LDSDS were verified (Figure 4) in another independent patient cohorts. To observe the sensitivity and specificity of these mRNAs, ROC curve analysis was conducted to differentiating DHAS and LDSDS. The AUC of the three genes was 0.566, 0.372 and 0.702, respectively (Figure 5-A). The mRNA panel with the three mRNAs from the multivariate logistic regression model demonstrated high accuracy (AUC = 0.835) in the differentiation of DHAS and LDSDS (Figure 5-B). The result indicated that the three genes may be involved in DHAS and LDSDS differentiation in HBC. *PNP*, a key enzyme in the purine salvage pathway, is located primarily in the cytoplasm of endothelial cells, Kupffer cells, and hepatocytes and is released into hepatic sinusoids during necrosis [25] there are studies showed the value of *PNP* as a marker of liver damage in rodents [26]. *AQP7* are transmembrane proteins that belong to the subset of aquaglyceroporins. The lack of *AQP7* in both cases resulted in an increased accumulation of glycerol within the adipocytes leading to adipocyte hypertrophy [27]. A large-scale screening study [28] has been reported that *PSMD2* offers a growth advantage to NIH3T3 cells under certain conditions, which was known as a collagen-producing fibroblast cell. Therefore, *PSMD2* might be involved in the generation of liver fibrosis. Epidemiological studies [29, 30] have reported that HBC develop to HCC often following with TCM ZHENG changes from excess to deficiency. In this study, *AQP7* and *PSMD2* expression in LDSDS was significantly higher than that in DHAS. We speculated that both HBC prognosis and HCC morbidity in LDSDS patients may be more than that in DHAS patients. A future study to elucidate how such molecular consequences are mediated in *PNP*, *AQP7* and *PSMD2*-involed relationship between HBC and TCM ZHENG is warranted. It indicated a possibility of distinguishing DHAS and LDSDS by the expressions of *PNP*, *AQP7* and *PSMD2* their combination.

Furthermore, we performed GO function enrichment analysis to understand the different functions of DHAS and LDSDS. The results indicated that there are similarities of gene functions in DHAS and LDSDS, they all mainly involved in system development, anatomical structure development and so on. Otherwise, the DHAS was most likely related to cellular process when the functions overrepresented by LDSDS samples most related to the response to stimulus (Figure 6). Previous studies [31, 32] showed that the central neurobiological mechanism of LDSDS closely correlates to the hypothalamic-pituitary-adrenal axis, brain-gut axis, myriad central neurotropic factors, neurotransmitters and neuropeptides involving in many encephalic regions such as the hypothalamus, hippocampus, cortex, amygdale, etc. This funding was consisting with our GO analysis, which related to the chronic stress.

The limitations of this study are as follows. (1) A small sample size limited the value and generalization of the results; a larger population study should be further considered in both test group and validation group. (2) Only two typical ZHENGs in HBC were discussed in this study. Other ZHENGs types of HBC required for further research. (3) Since the gene regulation network is complicate and remaining unclear in HBV-caused chronic diseases, the mechanism of selected genes needs further study.

## Conclusions

In this study, we demonstrated the transcriptional profiles, differential co-expression genes and their functional properties for ZHENG differentiation in HBC patients. The results suggested that there are particular transcriptional profiles, genes co-expressions patterns and their functional properties of DHAS and LDSDS, and several interesting genes including *PNP*, *AQP7*, *PSMD2* and their combination may be involved in ZHENG differentiation of DHAS and LDSDS in HBC. Although further investigation with larger number of samples and other type ZHENGs is requested, these results supported the hypothesis that transcriptional profiles may be used to the ZHENG differentiation in HBC.

## Electronic supplementary material

Additional file 1: **DEGs of DHAS and LDSDS.** (XLSX 375 KB)

## Abbreviations

TCM: traditional Chinese medicine
HBC: hepatitis B-caused cirrhosis
DHAS: dampness-heat accumulation syndrome
LDSDS: liver depression and spleen deficiency syndrome
DEGs: differentially expressed genes
GeneRelNet: Gene-Regulatory-Networks
PNP: purine nucleoside phosphorylase
AQP7: aquaporin 7
PSMD2: proteasome 26S subunit, non-ATPase 2
HBV: Chronic hepatitis B virus
HCC: hepatocellular carcinoma
CHB: chronic hepatitis B
HBC: hepatitis B-caused cirrhosis
ALT: alanine aminotransferase
AST: aspartate aminotransferase
AFP: Alpha Fetal Protein
HA: Hyaluronic Acid
TBIL: total bilirubin
DBIL: direct bilirubin
GGT: gamma glutamyl transferase
ALb: albumin
GO: Gene Ontology
ROC: Receiver-operator characteristic.
